# *catena*-Poly[[(1,10-phenanthroline-κ^2^ *N*,*N*′)lanthanum(III)]-μ-(5-bromo-2-hy­droxy­benzoato)-κ^2^ *O* ^1^:*O* ^1′^-di-μ-chlorido]

**DOI:** 10.1107/S1600536812025500

**Published:** 2012-06-13

**Authors:** Wen-Qing Zhu, Jin-Ping Wang, Yu-Kun Yuan, Zhuo Li

**Affiliations:** a College of Environment and Chemical Engineering, Xi’an Polytechnic University, Xi’an, Shaanxi 710048, People’s Republic of China

## Abstract

In the title complex, [La(C_7_H_4_BrO_3_)Cl_2_(C_12_H_8_N_2_)]_*n*_, the La^III^ ion is eight-coordinated by two carboxyl­ate O atoms from two 5-bromo­salicylate ligands, two N atoms from a chelating 1,10-phenanthroline ligand and four bridging Cl atoms in a distorted square-anti­prismatic geometry. The La^III^ ions are linked by bridging carboxyl­ate groups and chloride anions into a chain along [100]. An intra­molecular O—H⋯O hydrogen bond is formed in the 5-bromo­salicylate ligand. π–π inter­actions between the pyridine and benzene rings and between the benzene rings are observed [centroid–centroid distances = 3.794 (5) and 3.804 (4) Å].

## Related literature

For background to rare earth carboxyl­ates, see: Ali *et al.* (2004 ▶); Costes *et al.* (2002 ▶); Kaur *et al.* (2010 ▶); Yin & Sun (2004 ▶). For complexes with salicylate ligands, see: Hu *et al.* (2005 ▶); Yin *et al.* (2004 ▶).
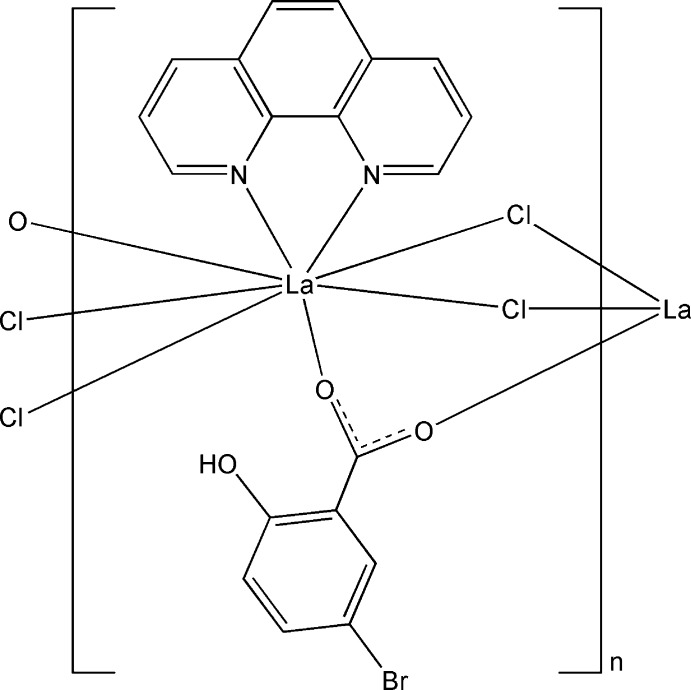


## Experimental

### 

#### Crystal data

[La(C_7_H_4_BrO_3_)Cl_2_(C_12_H_8_N_2_)]
*M* *_r_* = 606.02
Orthorhombic, 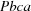

*a* = 8.2957 (10) Å
*b* = 22.104 (3) Å
*c* = 22.110 (3) Å
*V* = 4054.3 (9) Å^3^
*Z* = 8
Mo *K*α radiation
μ = 4.36 mm^−1^
*T* = 296 K
0.30 × 0.24 × 0.14 mm

#### Data collection

Bruker APEXII CCD diffractometer
Absorption correction: multi-scan (*SADABS*; Sheldrick, 1996 ▶) *T*_min_ = 0.352, *T*_max_ = 0.584
19025 measured reflections
3598 independent reflections
2849 reflections with *I* > 2σ(*I*)
*R*_int_ = 0.065

#### Refinement

*R*[*F* ^2^ > 2σ(*F* ^2^)] = 0.034
*wR*(*F* ^2^) = 0.125
*S* = 1.00
3598 reflections
254 parameters
H-atom parameters constrained
Δρ_max_ = 1.20 e Å^−3^
Δρ_min_ = −1.34 e Å^−3^

### 

Data collection: *APEX2* (Bruker, 2007 ▶); cell refinement: *SAINT* (Bruker, 2007 ▶); data reduction: *SAINT*; program(s) used to solve structure: *SHELXS97* (Sheldrick, 2008 ▶); program(s) used to refine structure: *SHELXL97* (Sheldrick, 2008 ▶); molecular graphics: *DIAMOND* (Brandenburg, 1999 ▶) and *Mercury* (Macrae *et al.*, 2006 ▶); software used to prepare material for publication: *SHELXTL* (Sheldrick, 2008 ▶).

## Supplementary Material

Crystal structure: contains datablock(s) I, global. DOI: 10.1107/S1600536812025500/hy2550sup1.cif

Structure factors: contains datablock(s) I. DOI: 10.1107/S1600536812025500/hy2550Isup2.hkl

Additional supplementary materials:  crystallographic information; 3D view; checkCIF report

## Figures and Tables

**Table 1:** Hydrogen-bond geometry (Å, °)

*D*—H⋯*A*	*D*—H	H⋯*A*	*D*⋯*A*	*D*—H⋯*A*
O3—H3⋯O2	0.82	1.93	2.588 (6)	137

**Fig. 1.**
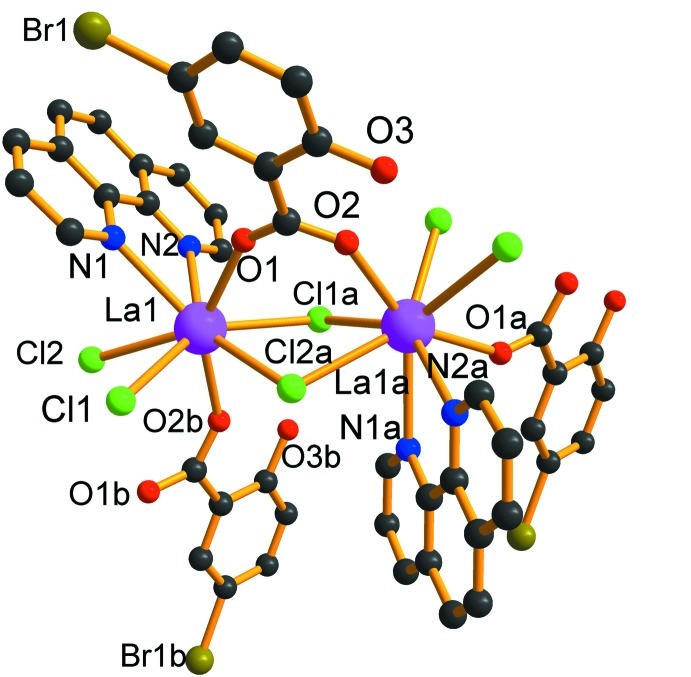
The asymmetric unit of the title compound. Displacement ellipsoids are drawn at the 30% probability level. H atoms have been omitted for clarity. [Symmetry codes: (a) x + 1/2, -y + 1/2, -z + 1; (b) x - 1/2, -y + 1/2, -z + 1.]

**Fig. 2.**
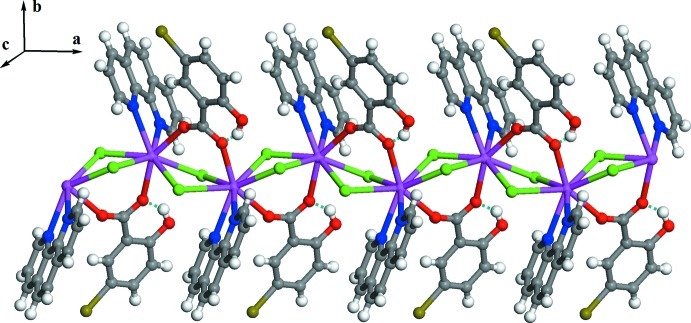
The one-dimensional chain structure in the title compound. Hydrogen bonds are shown as dashed lines.

## supplementary crystallographic information

### Comment

Over the past years, much attention has been paid to the rare earth carboxylates, owing to their novel structures and potential applications in a wide range of materials science, such as superconductor, magnetic materials and luminescent probes (Ali *et al.*, 2004; Costes *et al.*, 2002; Kaur *et al.*, 2010; Yin & Sun, 2004). Because salicylic acid and its derivatives have been known for a long time to possess anti-inflammatory activity and have also been considered of interest from a structural point of view (Hu *et al.*, 2005; Yin *et al.*, 2004), a new lanthanum bromosalicylate complex was synthesized and its crystal structure is reported here.

### Experimental

An ethanol solution (5 ml) of LaCl_3_.6H_2_O (0.5 mmol, 0.177 g) was added dropwise to an ethanol solution (5 ml) of 5-bromosalicylic acid (0.5 mmol, 0.109 g) under stirring and then an ethanol solution (5 ml) of 1,10-phenanthroline (0.5 mmol, 0.084 g) was added dropwise. The mixture was stirred for about 13 min at room temperature and sealed in a Teflon-lined stainless autoclave and heated to 120°C for 60 h. After filtered, colorless flake-shaped crystals were obtained.

### Refinement

H atoms were positioned geometrically and refined as riding atoms, with O—H = 0.82 and C—H = 0.93 Å and with *U*_iso_(H) = 1.2*U*_eq_(C) and 1.5*U*_eq_(O). The highest residual electron density was found 1.20 Å from Br1 atom and the deepest hole 1.34 Å from La1 atom.

### Crystal data

**Table:**

[La(C_7_H_4_BrO_3_)Cl_2_(C_12_H_8_N_2_)]	*F*(000) = 2320
*M**_r_* = 606.02	*D*_x_ = 1.986 Mg m^−^^3^
Orthorhombic, *P**b**c**a*	Mo *K*α radiation, λ = 0.71073 Å
Hall symbol: -P 2ac 2ab	Cell parameters from 3026 reflections
*a* = 8.2957 (10) Å	θ = 2.6–24.6°
*b* = 22.104 (3) Å	µ = 4.36 mm^−^^1^
*c* = 22.110 (3) Å	*T* = 296 K
*V* = 4054.3 (9) Å^3^	Flake, colorless
*Z* = 8	0.30 × 0.24 × 0.14 mm

### Data collection

**Table:**

Bruker APEXII CCD diffractometer	3598 independent reflections
Radiation source: fine-focus sealed tube	2849 reflections with *I* > 2σ(*I*)
Graphite monochromator	*R*_int_ = 0.065
φ and ω scans	θ_max_ = 25.1°, θ_min_ = 1.8°
Absorption correction: multi-scan (*SADABS*; Sheldrick, 1996)	*h* = −9→7
*T*_min_ = 0.352, *T*_max_ = 0.584	*k* = −26→26
19025 measured reflections	*l* = −26→26

### Refinement

**Table:**

Refinement on *F*^2^	Primary atom site location: structure-invariant direct methods
Least-squares matrix: full	Secondary atom site location: difference Fourier map
*R*[*F*^2^ > 2σ(*F*^2^)] = 0.034	Hydrogen site location: inferred from neighbouring sites
*wR*(*F*^2^) = 0.125	H-atom parameters constrained
*S* = 1.00	*w* = 1/[σ^2^(*F*_o_^2^) + (0.080*P*)^2^ + 0.7837*P*] where *P* = (*F*_o_^2^ + 2*F*_c_^2^)/3
3598 reflections	(Δ/σ)_max_ = 0.002
254 parameters	Δρ_max_ = 1.20 e Å^−^^3^
0 restraints	Δρ_min_ = −1.34 e Å^−^^3^

### Special details

**Table:**

Geometry. All e.s.d.'s (except the e.s.d. in the dihedral angle between two l.s. planes) are estimated using the full covariance matrix. The cell e.s.d.'s are taken into account individually in the estimation of e.s.d.'s in distances, angles and torsion angles; correlations between e.s.d.'s in cell parameters are only used when they are defined by crystal symmetry. An approximate (isotropic) treatment of cell e.s.d.'s is used for estimating e.s.d.'s involving l.s. planes.
Refinement. Refinement of *F*^2^ against ALL reflections. The weighted *R*-factor *wR* and goodness of fit *S* are based on *F*^2^, conventional *R*-factors *R* are based on *F*, with *F* set to zero for negative *F*^2^. The threshold expression of *F*^2^ > σ(*F*^2^) is used only for calculating *R*-factors(gt) *etc*. and is not relevant to the choice of reflections for refinement. *R*-factors based on *F*^2^ are statistically about twice as large as those based on *F*, and *R*- factors based on ALL data will be even larger.

### Fractional atomic coordinates and isotropic or equivalent isotropic displacement parameters (Å^2^)

**Table:**

	*x*	*y*	*z*	*U*_iso_*/*U*_eq_	
La1	0.78367 (4)	0.242463 (15)	0.537359 (15)	0.02456 (15)	
Br1	0.86777 (13)	−0.05046 (5)	0.66282 (5)	0.0877 (4)	
Cl1	0.57034 (19)	0.18099 (7)	0.45631 (7)	0.0352 (4)	
Cl2	0.46216 (18)	0.27765 (8)	0.57361 (7)	0.0365 (4)	
N1	0.6683 (7)	0.1654 (3)	0.6202 (2)	0.0401 (14)	
N2	0.8088 (7)	0.2724 (3)	0.6548 (2)	0.0396 (14)	
O1	0.9829 (5)	0.16436 (19)	0.5594 (2)	0.0389 (11)	
O2	1.2182 (5)	0.1607 (2)	0.5117 (2)	0.0423 (12)	
O3	1.3579 (7)	0.0573 (2)	0.4954 (3)	0.0615 (15)	
H3	1.3282	0.0899	0.4818	0.092*	
C1	0.5984 (9)	0.1136 (3)	0.6047 (4)	0.0510 (19)	
H1	0.5907	0.1048	0.5637	0.061*	
C2	0.5357 (10)	0.0711 (4)	0.6451 (4)	0.067 (2)	
H2	0.4911	0.0349	0.6317	0.080*	
C3	0.5428 (11)	0.0852 (5)	0.7052 (4)	0.075 (3)	
H3A	0.5001	0.0585	0.7334	0.090*	
C4	0.6131 (10)	0.1391 (4)	0.7249 (3)	0.059 (2)	
C5	0.6262 (12)	0.1591 (5)	0.7881 (3)	0.082 (3)	
H5	0.5853	0.1341	0.8184	0.098*	
C6	0.6954 (12)	0.2124 (5)	0.8043 (4)	0.077 (3)	
H6	0.7022	0.2228	0.8450	0.093*	
C7	0.7564 (11)	0.2516 (4)	0.7606 (4)	0.056 (2)	
C8	0.8293 (11)	0.3067 (4)	0.7754 (4)	0.065 (2)	
H8	0.8383	0.3182	0.8157	0.078*	
C9	0.8855 (11)	0.3424 (4)	0.7323 (4)	0.062 (2)	
H9	0.9332	0.3792	0.7419	0.074*	
C10	0.8721 (9)	0.3240 (3)	0.6727 (3)	0.0482 (19)	
H10	0.9107	0.3501	0.6431	0.058*	
C11	0.7488 (9)	0.2351 (3)	0.6985 (3)	0.0417 (17)	
C12	0.6755 (8)	0.1795 (3)	0.6812 (3)	0.0417 (17)	
C13	1.0177 (9)	−0.0158 (3)	0.6085 (3)	0.0481 (18)	
C14	1.1393 (10)	−0.0516 (3)	0.5865 (4)	0.056 (2)	
H14	1.1465	−0.0922	0.5971	0.067*	
C15	1.2497 (11)	−0.0257 (4)	0.5485 (4)	0.060 (2)	
H15	1.3323	−0.0496	0.5331	0.072*	
C16	1.2430 (9)	0.0348 (3)	0.5321 (3)	0.0442 (18)	
C17	1.1162 (8)	0.0710 (3)	0.5553 (3)	0.0316 (14)	
C18	1.0040 (8)	0.0436 (3)	0.5932 (3)	0.0397 (16)	
H18	0.9185	0.0662	0.6083	0.048*	
C19	1.1035 (8)	0.1364 (3)	0.5407 (3)	0.0352 (15)	

### Atomic displacement parameters (Å^2^)

**Table:**

	*U*^11^	*U*^22^	*U*^33^	*U*^12^	*U*^13^	*U*^23^
La1	0.0211 (2)	0.0260 (2)	0.0266 (2)	0.00183 (13)	−0.00042 (14)	0.00254 (13)
Br1	0.0732 (7)	0.0716 (7)	0.1182 (9)	−0.0105 (5)	0.0171 (6)	0.0530 (6)
Cl1	0.0248 (8)	0.0350 (9)	0.0459 (9)	0.0010 (6)	−0.0002 (7)	−0.0084 (7)
Cl2	0.0264 (8)	0.0551 (10)	0.0282 (8)	0.0097 (7)	−0.0002 (6)	−0.0023 (7)
N1	0.031 (3)	0.045 (3)	0.044 (3)	0.003 (3)	0.000 (3)	0.012 (3)
N2	0.032 (3)	0.052 (4)	0.035 (3)	0.011 (3)	−0.001 (2)	0.004 (3)
O1	0.034 (3)	0.039 (3)	0.043 (3)	0.012 (2)	0.009 (2)	0.012 (2)
O2	0.031 (3)	0.032 (3)	0.064 (3)	0.001 (2)	0.010 (2)	0.018 (2)
O3	0.051 (3)	0.045 (3)	0.088 (4)	0.017 (3)	0.032 (3)	0.013 (3)
C1	0.051 (5)	0.048 (4)	0.054 (4)	−0.001 (4)	−0.003 (4)	0.013 (4)
C2	0.059 (6)	0.061 (5)	0.081 (6)	−0.013 (4)	−0.005 (5)	0.032 (5)
C3	0.059 (6)	0.095 (7)	0.071 (6)	−0.014 (5)	0.005 (5)	0.050 (5)
C4	0.046 (5)	0.077 (6)	0.054 (5)	0.007 (4)	0.011 (4)	0.030 (4)
C5	0.077 (7)	0.137 (10)	0.032 (5)	0.018 (7)	0.012 (4)	0.044 (5)
C6	0.076 (7)	0.111 (9)	0.044 (5)	0.008 (6)	−0.004 (5)	0.010 (6)
C7	0.057 (6)	0.085 (7)	0.027 (4)	0.026 (4)	0.002 (4)	0.010 (4)
C8	0.074 (6)	0.079 (7)	0.043 (5)	0.020 (5)	−0.020 (4)	−0.022 (5)
C9	0.071 (6)	0.068 (6)	0.045 (5)	0.013 (5)	−0.016 (4)	−0.011 (4)
C10	0.046 (5)	0.055 (5)	0.043 (4)	0.008 (4)	−0.002 (3)	−0.013 (3)
C11	0.031 (4)	0.058 (5)	0.036 (4)	0.016 (3)	0.000 (3)	0.005 (3)
C12	0.023 (3)	0.064 (5)	0.038 (4)	0.017 (3)	0.007 (3)	0.018 (3)
C13	0.047 (5)	0.039 (4)	0.058 (5)	−0.008 (3)	−0.002 (4)	0.014 (3)
C14	0.060 (5)	0.032 (4)	0.076 (6)	0.000 (4)	0.000 (5)	0.010 (4)
C15	0.063 (5)	0.033 (4)	0.084 (6)	0.020 (4)	0.000 (5)	−0.004 (4)
C16	0.038 (4)	0.032 (4)	0.062 (5)	0.004 (3)	−0.001 (4)	−0.004 (3)
C17	0.030 (4)	0.024 (3)	0.041 (4)	0.002 (3)	−0.007 (3)	0.002 (3)
C18	0.034 (4)	0.036 (4)	0.048 (4)	0.001 (3)	−0.001 (3)	0.010 (3)
C19	0.032 (4)	0.037 (4)	0.037 (4)	0.004 (3)	−0.007 (3)	0.002 (3)

### Geometric parameters (Å, º)

**Table:**

La1—O1	2.439 (4)	C3—H3A	0.9300
La1—O2^i^	2.461 (4)	C4—C12	1.415 (10)
La1—N1	2.678 (5)	C4—C5	1.469 (12)
La1—N2	2.687 (6)	C5—C6	1.359 (14)
La1—Cl1	2.8618 (16)	C5—H5	0.9300
La1—Cl2	2.8915 (16)	C6—C7	1.393 (14)
La1—Cl2^ii^	2.9001 (16)	C6—H6	0.9300
La1—Cl1^ii^	2.9219 (16)	C7—C8	1.397 (12)
Br1—C13	1.891 (7)	C7—C11	1.422 (11)
Cl1—La1^i^	2.9219 (16)	C8—C9	1.321 (12)
Cl2—La1^i^	2.9001 (16)	C8—H8	0.9300
N1—C1	1.328 (9)	C9—C10	1.383 (10)
N1—C12	1.386 (9)	C9—H9	0.9300
N2—C10	1.317 (9)	C10—H10	0.9300
N2—C11	1.365 (10)	C11—C12	1.424 (11)
O1—C19	1.246 (8)	C13—C18	1.361 (9)
O2—C19	1.266 (8)	C13—C14	1.369 (10)
O2—La1^ii^	2.461 (4)	C14—C15	1.368 (12)
O3—C16	1.348 (9)	C14—H14	0.9300
O3—H3	0.8200	C15—C16	1.386 (12)
C1—C2	1.395 (10)	C15—H15	0.9300
C1—H1	0.9300	C16—C17	1.418 (9)
C2—C3	1.365 (13)	C17—C18	1.392 (9)
C2—H2	0.9300	C17—C19	1.485 (8)
C3—C4	1.396 (13)	C18—H18	0.9300
			
O1—La1—O2^i^	148.59 (15)	C3—C4—C12	118.5 (8)
O1—La1—N1	69.84 (15)	C3—C4—C5	125.7 (8)
O2^i^—La1—N1	140.90 (16)	C12—C4—C5	115.7 (9)
O1—La1—N2	85.91 (16)	C6—C5—C4	122.8 (8)
O2^i^—La1—N2	103.23 (17)	C6—C5—H5	118.6
N1—La1—N2	61.55 (19)	C4—C5—H5	118.6
O1—La1—Cl1	102.01 (12)	C5—C6—C7	120.7 (9)
O2^i^—La1—Cl1	90.03 (11)	C5—C6—H6	119.6
N1—La1—Cl1	84.55 (13)	C7—C6—H6	119.6
N2—La1—Cl1	140.34 (13)	C6—C7—C8	122.5 (9)
O1—La1—Cl2	139.46 (11)	C6—C7—C11	119.6 (9)
O2^i^—La1—Cl2	71.62 (11)	C8—C7—C11	117.8 (8)
N1—La1—Cl2	69.63 (12)	C9—C8—C7	120.4 (8)
N2—La1—Cl2	74.78 (12)	C9—C8—H8	119.8
Cl1—La1—Cl2	74.39 (4)	C7—C8—H8	119.8
O1—La1—Cl2^ii^	73.42 (11)	C8—C9—C10	118.8 (8)
O2^i^—La1—Cl2^ii^	82.71 (12)	C8—C9—H9	120.6
N1—La1—Cl2^ii^	131.57 (13)	C10—C9—H9	120.6
N2—La1—Cl2^ii^	144.64 (13)	N2—C10—C9	125.0 (8)
Cl1—La1—Cl2^ii^	73.33 (4)	N2—C10—H10	117.5
Cl2—La1—Cl2^ii^	138.31 (4)	C9—C10—H10	117.5
O1—La1—Cl1^ii^	81.30 (11)	N2—C11—C7	120.9 (8)
O2^i^—La1—Cl1^ii^	72.36 (11)	N2—C11—C12	119.0 (7)
N1—La1—Cl1^ii^	128.79 (13)	C7—C11—C12	120.0 (7)
N2—La1—Cl1^ii^	75.41 (13)	N1—C12—C4	120.5 (7)
Cl1—La1—Cl1^ii^	143.93 (3)	N1—C12—C11	118.4 (6)
Cl2—La1—Cl1^ii^	125.57 (5)	C4—C12—C11	121.1 (7)
Cl2^ii^—La1—Cl1^ii^	73.37 (4)	C18—C13—C14	122.0 (7)
La1—Cl1—La1^i^	101.44 (5)	C18—C13—Br1	119.5 (6)
La1—Cl2—La1^i^	101.25 (5)	C14—C13—Br1	118.4 (5)
C1—N1—C12	117.6 (6)	C15—C14—C13	118.0 (7)
C1—N1—La1	121.9 (5)	C15—C14—H14	121.0
C12—N1—La1	120.4 (4)	C13—C14—H14	121.0
C10—N2—C11	117.0 (7)	C14—C15—C16	122.4 (8)
C10—N2—La1	122.4 (5)	C14—C15—H15	118.8
C11—N2—La1	120.6 (5)	C16—C15—H15	118.8
C19—O1—La1	146.0 (4)	O3—C16—C15	119.0 (7)
C19—O2—La1^ii^	139.2 (4)	O3—C16—C17	122.2 (6)
C16—O3—H3	109.5	C15—C16—C17	118.7 (8)
N1—C1—C2	125.3 (8)	C18—C17—C16	117.9 (6)
N1—C1—H1	117.4	C18—C17—C19	120.5 (6)
C2—C1—H1	117.4	C16—C17—C19	121.7 (6)
C3—C2—C1	116.9 (8)	C13—C18—C17	120.9 (7)
C3—C2—H2	121.5	C13—C18—H18	119.5
C1—C2—H2	121.5	C17—C18—H18	119.5
C2—C3—C4	121.1 (8)	O1—C19—O2	124.2 (6)
C2—C3—H3A	119.5	O1—C19—C17	117.9 (6)
C4—C3—H3A	119.5	O2—C19—C17	117.9 (6)
			
O1—La1—Cl1—La1^i^	161.75 (11)	C2—C3—C4—C5	179.5 (9)
O2^i^—La1—Cl1—La1^i^	−47.48 (12)	C3—C4—C5—C6	−179.6 (10)
N1—La1—Cl1—La1^i^	93.73 (13)	C12—C4—C5—C6	−0.9 (14)
N2—La1—Cl1—La1^i^	63.6 (2)	C4—C5—C6—C7	0.9 (16)
Cl2—La1—Cl1—La1^i^	23.40 (5)	C5—C6—C7—C8	−179.7 (10)
Cl2^ii^—La1—Cl1—La1^i^	−129.86 (6)	C5—C6—C7—C11	−1.2 (14)
Cl1^ii^—La1—Cl1—La1^i^	−106.56 (8)	C6—C7—C8—C9	180.0 (9)
O1—La1—Cl2—La1^i^	−113.89 (18)	C11—C7—C8—C9	1.5 (13)
O2^i^—La1—Cl2—La1^i^	71.80 (13)	C7—C8—C9—C10	−0.6 (13)
N1—La1—Cl2—La1^i^	−113.49 (14)	C11—N2—C10—C9	1.6 (11)
N2—La1—Cl2—La1^i^	−178.30 (15)	La1—N2—C10—C9	178.7 (6)
Cl1—La1—Cl2—La1^i^	−23.57 (5)	C8—C9—C10—N2	−1.0 (13)
Cl2^ii^—La1—Cl2—La1^i^	16.84 (4)	C10—N2—C11—C7	−0.5 (10)
Cl1^ii^—La1—Cl2—La1^i^	122.73 (5)	La1—N2—C11—C7	−177.7 (5)
O1—La1—N1—C1	−84.2 (5)	C10—N2—C11—C12	178.4 (6)
O2^i^—La1—N1—C1	104.1 (6)	La1—N2—C11—C12	1.2 (8)
N2—La1—N1—C1	179.4 (6)	C6—C7—C11—N2	−179.5 (8)
Cl1—La1—N1—C1	20.8 (5)	C8—C7—C11—N2	−0.9 (12)
Cl2—La1—N1—C1	96.1 (5)	C6—C7—C11—C12	1.6 (12)
Cl2^ii^—La1—N1—C1	−41.2 (6)	C8—C7—C11—C12	−179.9 (7)
Cl1^ii^—La1—N1—C1	−144.0 (5)	C1—N1—C12—C4	1.2 (9)
O1—La1—N1—C12	97.8 (5)	La1—N1—C12—C4	179.3 (5)
O2^i^—La1—N1—C12	−73.9 (5)	C1—N1—C12—C11	−179.5 (6)
N2—La1—N1—C12	1.4 (4)	La1—N1—C12—C11	−1.4 (8)
Cl1—La1—N1—C12	−157.2 (5)	C3—C4—C12—N1	−0.7 (11)
Cl2—La1—N1—C12	−81.9 (4)	C5—C4—C12—N1	−179.5 (7)
Cl2^ii^—La1—N1—C12	140.8 (4)	C3—C4—C12—C11	−179.9 (7)
Cl1^ii^—La1—N1—C12	38.0 (5)	C5—C4—C12—C11	1.3 (11)
O1—La1—N2—C10	112.4 (5)	N2—C11—C12—N1	0.2 (10)
O2^i^—La1—N2—C10	−37.2 (5)	C7—C11—C12—N1	179.1 (6)
N1—La1—N2—C10	−178.4 (6)	N2—C11—C12—C4	179.4 (6)
Cl1—La1—N2—C10	−143.7 (5)	C7—C11—C12—C4	−1.7 (11)
Cl2—La1—N2—C10	−103.6 (5)	C18—C13—C14—C15	0.5 (12)
Cl2^ii^—La1—N2—C10	58.9 (6)	Br1—C13—C14—C15	−178.3 (6)
Cl1^ii^—La1—N2—C10	30.3 (5)	C13—C14—C15—C16	0.5 (13)
O1—La1—N2—C11	−70.6 (5)	C14—C15—C16—O3	178.7 (8)
O2^i^—La1—N2—C11	139.9 (5)	C14—C15—C16—C17	−0.5 (13)
N1—La1—N2—C11	−1.3 (5)	O3—C16—C17—C18	−179.6 (7)
Cl1—La1—N2—C11	33.4 (6)	C15—C16—C17—C18	−0.4 (10)
Cl2—La1—N2—C11	73.4 (5)	O3—C16—C17—C19	−0.6 (11)
Cl2^ii^—La1—N2—C11	−124.0 (5)	C15—C16—C17—C19	178.6 (7)
Cl1^ii^—La1—N2—C11	−152.6 (5)	C14—C13—C18—C17	−1.4 (11)
O2^i^—La1—O1—C19	−32.9 (9)	Br1—C13—C18—C17	177.4 (5)
N1—La1—O1—C19	157.1 (8)	C16—C17—C18—C13	1.3 (10)
N2—La1—O1—C19	−141.8 (8)	C19—C17—C18—C13	−177.7 (6)
Cl1—La1—O1—C19	77.5 (8)	La1—O1—C19—O2	39.6 (12)
Cl2—La1—O1—C19	157.5 (7)	La1—O1—C19—C17	−142.6 (6)
Cl2^ii^—La1—O1—C19	9.2 (8)	La1^ii^—O2—C19—O1	−9.6 (11)
Cl1^ii^—La1—O1—C19	−65.9 (8)	La1^ii^—O2—C19—C17	172.6 (4)
C12—N1—C1—C2	−2.0 (11)	C18—C17—C19—O1	−5.8 (9)
La1—N1—C1—C2	179.9 (6)	C16—C17—C19—O1	175.2 (6)
N1—C1—C2—C3	2.1 (13)	C18—C17—C19—O2	172.2 (6)
C1—C2—C3—C4	−1.4 (14)	C16—C17—C19—O2	−6.8 (9)
C2—C3—C4—C12	0.8 (13)		

Symmetry codes: (i) *x*−1/2, −*y*+1/2, −*z*+1; (ii) *x*+1/2, −*y*+1/2, −*z*+1.

### Hydrogen-bond geometry (Å, º)

**Table:**

*D*—H···*A*	*D*—H	H···*A*	*D*···*A*	*D*—H···*A*
O3—H3···O2	0.82	1.93	2.588 (6)	137
